# Supplementary material for: Experimental Tests for Measuring Individual Attentional Characteristics in Songbirds
Source: Animals (Basel). 2021 Jul 29;11(8):2233. doi: 10.3390/ani11082233 (PMC8388455; doi:10.3390/ani11082233)
Supplement: Supplementary file 1 [file animals-11-02233-s001.zip › animals-1255122-supplementary/Table S1.pdf]

Table S1: Occurrences of glances and gazes during all attentional tests. Wilcoxon signed-rank test and N=10 for each test. ($\bar{X}$ = Mean; **S.E.** = Standard Error**; C.V.** = Coefficient of variation). SVAT = Social Visual Attention Test, VAT= Visual Attention Test, AAT = Auditory Attention Test. Mono=monocular, Bino= binocular, Stim= stimulus

		**Visual Attention**								**Auditory Attention**						
		S.V.A.T.								*A.A.T conspecific stim*						
**Social Attention**		**Glance**		**Gaze**		**T**	**p-value**	**Sign**								
		$\bar{X}$±S.E.	***C.V.***	***$\bar{X}$±S.E.***	***C.V.***											
	**Max.**	31.1±4	43	4.3±0.9	70.2	0	=0.006	**		**Before Stim**		**After Stim**		**T**	**p-value**	**Sign**
	**Min.**	16.2±3.1	62	6.1±1.5	70.2	3.5	=0.01	*		***$\bar{X}$±S.E.***	***C.V.***	***$\bar{X}$±S.E.***	***C.V.***			
		**Mono.**		**Bino.**		**T**	**p-value**	**Sign**	**Glance**	42.7±4.9	38.1	40.3±5.5	36.4	11	+0.09	NS
		$\bar{X}$±S.E.	***C.V.***	***$\bar{X}$±S.E.***	***C.V.***				**Gaze**	6.1±1.9	103.8	23.7±2.0	27.5	0	0.006	**
	**Glance**	19.2±2.8	46.2	11.8±1.9	52.2	8	=0.04	*								
	**Gaze**	3.9±1	82.4	0.4±0.2	174.8	8	=0.04	*								
		V.A.T.								*A.A.T. heterospecific stim*						
**Non-Social Attention**		**Before Stim**		**During Stim**		**T**	**p-value**	**Sign**		**Before Stim**		**After Stim**		**T**	**p-value**	**Sign**
		$\bar{X}$±S.E.	***C.V.***	***$\bar{X}$±S.E.***	***C.V.***					***$\bar{X}$±S.E.***	***C.V.***	***$\bar{X}$±S.E.***	***C.V.***			
	**Glance**	63.8±11	57.2	75.1±7.8	34.6	15	=0.2	NS	**Glance**	46.7±2.6	18.5	74.5±4.7	21.02	1	=0.007	**
	**Gaze**	16.5±4.1	83.1	33.6±5.9	58.7	5	=0.02	*	**Gaze**	6±1.8	98.4	8±2.8	114.4	24	=.7	NS
