# Supplementary material for: Experimental Tests for Measuring Individual Attentional Characteristics in Songbirds
Source: Animals (Basel). 2021 Jul 29;11(8):2233. doi: 10.3390/ani11082233 (PMC8388455; doi:10.3390/ani11082233)
Supplement: Supplementary file 1 [file animals-11-02233-s001.zip › animals-1255122-supplementary/Table S2.pdf]

Table S2: Total gaze duration (s) during the attention tests. Wilcoxon signed-rank test and N=10 for each test. ($\bar{X}$ **=** Mean; **S.E.** = Standard Error**; C.V.** = Coefficient of variation); SVAT = Social Visual Attention Test, VAT= Visual Attention Test, AAT = Auditory Attention Test.

	**Visual Attention**							**Auditory Attention**						
	S.V.A.T.							*A.A.T conspecific stim*						
**Social Attention**	**Max.**		**Min.**		**T**	**p-value**	**Sign**	**Before Stim**		**After Stim**		**T**	**p-value**	**Sign**
	$\bar{X}$±S.E.	***C.V.***	***$\bar{X}$±S.E.***	***C.V.***				***$\bar{X}$±S.E.***	***C.V.***	***$\bar{X}$±S.E.***	***C.V.***			
	9.6±2.2	78	13.1±3.6	90.4	12	=0.2	NS	19.9±6.9	114.4	84.3±10.1	39.7	0	=0.006	**
	**Mono.**		**Bino.**		**T**	**p-value**	**Sign**							
	$\bar{X}$±S.E.	***C.V.***	***$\bar{X}$±S.E.***	***C.V.***										
	8.8±2.5	88.3	0.8±0.4	174.8	4	=0.02	*							
	V.A.T.							A.A.T. heterospecific stim						
**Non-Social Attention**	Before Stim		During Stim		**T**	**p-value**	**Sign**	**Before Stim**		**After Stim**		**T**	**p-value**	**Sign**
	$\bar{X}$±S.E.	***C.V.***	***$\bar{X}$±S.E.***	***C.V.***				***$\bar{X}$±S.E.***	***C.V.***	***$\bar{X}$±S.E.***	***C.V.***			
	15.5±4.9	104	53±9.9	61.7	1	=0.007	**	15.5±5.6	122.3	23.7±8.6	120.9	23	=0.6	NS
